# Motivation, self-determination, and long-term weight control

**DOI:** 10.1186/1479-5868-9-22

**Published:** 2012-03-02

**Authors:** Pedro J Teixeira, Marlene N Silva, Jutta Mata, António L Palmeira, David Markland

**Affiliations:** 1 Faculty of Human Kinetics, Technical University of Lisbon, Portugal; 2 University Lusófona of Humanities and Technologies, Lisbon, Portugal; 3 School of Sport, Health and Exercise Sciences, Bangor University, UK

## Abstract

This article explores the topics of motivation and self-regulation in the context of weight management and related behaviors. We focus on the role of a *qualitative *approach to address motivation - not only considering the level but also type of motivation - in weight control and related behaviors. We critically discuss the operationalization of motivation in current weight control programs, present a complementary approach to understanding motivation based on self-determination theory, and review empirical findings from weight control studies that have used self-determination theory measures and assessed their association with weight outcomes. Weight loss studies which used Motivational Interviewing (MI) are also reviewed, considering MI's focus on enhancing internal motivation. We hypothesize that current weight control interventions may have been less successful with weight maintenance in part due to their relative disregard of qualitative dimensions of motivation, such as level of perceived autonomy, often resulting in a motivational disconnect between weight loss and weight-related behaviors. We suggest that if individuals fully endorse weight loss-related behavioral goals and feel not just competent but also autonomous about reaching them, as suggested by self-determination theory, their efforts are more likely to result in long-lasting behavior change.

## Introduction

The recent increase in obesity is undoubtedly rooted in powerful environmental changes, which exert constant pressure, or at the least that make it increasingly easy for individuals to lead predominantly sedentary lives and eat high-energy dense foods in excess [1,2]. Under these conditions, and given limited research resources, some may question whether it is justifiable or useful to continue to study psychological and other self-regulatory features of the behaviors involved in weight control, such as motivation, attitudes, goals, and skills around relevant behaviors? While stronger policy measures to halt the "obesity epidemic" (e.g., [3]) may prove decisive in effectively fighting obesity at a population level, major environmental changes will take time to be implemented and are still in the early stages of effectiveness testing (e.g., [4]). Meanwhile, overweight and obese persons are living their lives in the *present *environment and, in one-on-one sessions with health professionals or as part of community or commercial group programs, seek more effective solutions and ask for advice on how to deal with their excess weight [5]. This is the main rationale to continue studying and improving so-called *individual-level *interventions. They exist in real life and influence the experiences of a large number of people. Additionally, the potential for these interventions to be effectively translated and implemented in mass scale (e.g., [6-8]) has not yet been fully tested and should not, in our view, be underestimated when considering population behavior change.

### Motivation in current weight control programs

Few people, especially those treating or counseling persons with obesity would argue against the importance of motivation as a predictor of treatment success. A lack of motivation leading to poor adherence has been presented as a rationale for including MI in weight control programs [9] and "internal motivation to lose weight" and "self-motivation" have been identified as predictors of successful weight control in previous review articles [10,11]. Surprisingly, in a recent review of obesity treatments, Powell et al. [12] concluded that successful lifestyle interventions "*used a variety of behavioral techniques to achieve goals, including self-monitoring, modeling, environmental restructuring, as well as group and individual support*. (p.242). Motivation is notably absent from this list. State-of-the-art behavioral interventions to reduce overweight or obesity (e.g., [13-15]) also offer limited detail into specific motivational mechanisms selected to influence behavior change. Traditionally, researchers in these trials appeared to have been primarily concerned with increasing and maintaining motivation *level*. This is reflected in statements such as "(using strategies)... to *increase *motivation." (p. 742, italics added), or "*Sustaining *motivation for behavior change is a key focus..." (p.743, italics added) [14]. Motivation was also expected to *decrease *for some people, at which time interventions needed to be prepared for "boosting" motivation (level) in participants with adherence issues.

Reducing motivation to its quantitative dimension could be an important limiting factor in current weight loss interventions. For example, very different events (e.g., a practitioner's firm instruction during a consultation, a personal decision after a life-threatening event, etc.) can lead people to initiate the same course of action, such as joining a weight loss program, potentially with no detectable difference in *how much *they want to lose weight. Similarly, motives such as wanting to lose weight to improve physical attractiveness or reduce body size/shape dissatisfaction may carry different implications during treatment from joining a weight loss program primarily to improve health or to learn how to engage the family in regular physical activity. Moreover, the source and nature of motivation for weight loss could markedly shift during the course of treatment. Although these questions have not been systematically investigated in current weight control interventions, we believe that examining the *nature *of goals and the *quality *of motivation behind the desire to lose weight can prove useful at various junctures of the weight management process, beyond considering the *amount *of a person's motivational impulse. For example, what is the personal *meaning *(or *meanings*) associated with participating in regular exercise, adopting a new dietary regimen, or achieving a reduced body weight? Why do some patients "lose motivation" and others do not, particularly after achieving weight loss and entering maintenance? Why do some participants feel that they have failed (and often abandon their efforts) when, despite having successfully improved their lifestyle and also their health, they did not lose a substantial amount of weight? What makes the difference between self-sustained and less consistent forms of motivation and associated behaviors? Although some authors have begun to differentiate intervention components into those that primarily address motivational facets associated with weight loss and those which target motivational aspects that foster weight loss maintenance [16,17], motivation in the obesity behavior change literature has almost exclusively been described in a one-dimensional, quantitative fashion that typically only focuses on *how *to achieve the outcome. A more sophisticated examination of the consequences of differences in the *quality *of motivation could help in understanding successful weight loss and eventually help design more effective interventions [18].

### Autonomous motivation: exploring the *why *and *what *of weight control behaviors

Within the context of self-determination theory, the role of personal autonomy in human agency is given primary attention as a characteristic of motivational quality [19,20]. Clearly differentiated from constructs such as independence or self-reliance, autonomy is related to the perceived origin of one's action or its *locus of causality *[21]. That is, the extent to which a behavior or course of action is personally endorsed and engaged in with a sense of choice and volition (autonomous motivation), as opposed to being associated with a need to comply or with feelings of pressure and tension, often manifested in expressions like "I should", "I ought to", "I must", etc. In the latter case, motivation is described as being controlled, either by introjects the person has internalized (e.g., anchored in guilt or needed to maintain self-esteem) or by external contingencies such as incentives or expected negative consequences from the behavior [19].

Whenever individuals embark on a weight loss program, clearly they will have particular goals in mind associated with a reduced weight, whether these are to improve appearance, for health and fitness reasons, or to please others. Self-determination theory distinguishes between the content of goals or aspirations (e.g., social connectedness, personal growth, fame and fortune, physical attractiveness) and different regulatory reasons (to conform, to maintain self-esteem, to have fun) in that behavioral pursuits associated with more extrinsic goals, for instance, projecting an attractive image, tend to be regulated by more controlled reasons [22]. Conversely, more intrinsic goals (i.e., health, affiliation, personal growth) tend to be directly connected to the satisfaction of basic psychological needs [23] and are typically regulated by more autonomous forms of motivation [24]. Indeed, in self-determination theory, the concept of autonomy is central to understanding goal pursuit and why *not all goals are created equal *[25]. Autonomy (or self-determination) is understood as an innate and universal human psychological need, along with the needs for competence (effectance) and relatedness (belonging) with others. Feeling autonomous and volitional in one's pursuits, feeling effective and optimally challenged, and feeling meaningfully connected to others are held to have intrinsic value to the self and are essential for well-being and behavioral persistence.

Exploring the degree of autonomy associated with individuals' choices and actions provides a *qualitatively *sophisticated characterization of motivation, with potentially important implications for understanding and promoting behavior initiation and especially its maintenance. According to self-determination theory, the same behavioral goal (e.g., self-monitoring one's diet or exercise routine) can be enacted according to various regulatory/motivational features, from externally driven (e.g., to avoid criticism from a health professional), to partially internalized (introjected) regulation (e.g., "the people in my weight loss group all keep exercise diaries; I really feel like I should do it too"), all the way to autonomous regulation. (Note: Some authors refer to *autonomous regulation *as *autonomous self-regulation*, placing the emphasis on autonomy as a distinctive feature of the regulatory process and differentiated from more traditional views of self-regulation [26]^1^). In the case of autonomous regulation, an individual might decide to self-monitor because gathering information on progress has become personally important (identified regulation; e.g., "I have realized that charting my progress helps me stay motivated") or because it is an interesting activity per se (intrinsic motivation; e.g., "I've created my own exercise spreadsheet on the computer and enjoy filling it out after class and calculating how many calories I burned"). The key difference to be noted is the level of "choicefulness" and personal endorsement ("authorship") associated with the course of action, which can be applied to all behaviors associated with weight control. In the words of Ryan and colleagues, it's not only important to address "*toward what kind of goals does the counseling or therapy aim*" but also "*who does the steering*" [27] (p.6).

From a behavior change viewpoint, the internalization process, that is, the assimilation of the behavior into the self and taking responsibility for its regulation has critical functional significance since interventions can be built precisely to foster ownership by the participant of the new behavioral patterns and the development of self-motivated reasons to change, as opposed to fomenting continued reliance on external support, incentives, and/or oversight [27].

### Lasting weight control through the lens of self-determination theory

#### Promoting self-motivation vs. providing "continuous care"

In their review of obesity interventions, Powell et al. [12] suggest that *"the idea that a lifestyle intervention for obesity should occur for a discrete period of time, terminate, and then have lasting effects over the duration of one's lifetime is outmoded." *(p.243), asserting that these interventions are only successful as long as participants are in treatment. They also write that *"patients tell us, in a number of ways, that they need ongoing treatment" *[12] (p.243); in fact, a "continuous care" model for treating obesity is widely recommended (e.g. [28]). Despite the unlikelihood that large numbers of overweight and obese adults can ever be treated and supported *for life *by trained health care professionals in a cost-effective manner, the latter statement deserves a closer analysis. One possible interpretation is that, from the beginning of treatment, patients have internalized the message that their condition is to be dealt with by procedures and techniques essentially under the responsibility and "steering" of an external expert. If individuals generally expect to be told what to do in order to manage their condition (e.g., simply follow a dietary/exercise prescription or take a medication), this in itself could condition heteronomous (i.e., controlled) motivation from the start and promote an external locus of causality, particularly if therapeutic options are not presented and a clear rationale for each recommendation is not discussed. In contrast, from a self-determination theory perspective, lasting behavior change depends not on complying with external demands for change but rather on accepting the regulation of change as one's own. As stated by Ryan and Deci, "*it is integration within personality rather than behavior change per se that is the aim of an SDT-approach*..." [21] (p.188). This requires internalizing the regulation of relevant behaviors and integrating them with one's sense of self and one's values and goals, so they can become the basis of autonomous regulation. From this perspective, ongoing treatment is clearly not inevitable nor is it desirable. In fact, even if this would be scalable to effectively affect the millions of people struggling with obesity, accepting that regular reinforcements and "booster sessions" are needed for lasting behavior change denotes a clear focus on *external regulation *of behavior, which is typical of operant conditioning theory and classic behaviorism [27]. At the very least, one could argue that this focus is clearly not compatible with promoting self-motivation and autonomous regulation [19]; adopting a more conservative theoretical perspective, obesity researchers and clinicians might ultimately have to decide which approach brings about the best overall outcomes: one focused on continued external incentives or one unequivocally focused on developing self-motivation. Just as one example, financial incentives may promote short-term weight loss but appear ineffective in the long-term [29].

#### Reconsidering treatment outcomes

On a related note, the selection of outcomes in obesity treatment may also merit renewed attention. Currently, weight change is the most consistent result reported in weight loss trials and interventions are evaluated, and their relative efficacy compared, largely based on these parameters (e.g., percent weight change from baseline, number of subjects who achieve 5% or 10% weight loss, etc. [30]). From a self-determination theory perspective, because changes energized by autonomous vs. controlled motivation, including those experienced during weight control, are associated with different psychological consequences [25,31], selected psychological and behavioral outcomes might also have to be routinely considered successful. Two examples are (i) autonomous non-compliance, where a well-informed participant volitionally decides not to partake in behavior change despite professional advice [32], and (ii) maintenance of physical activity (or a healthful eating pattern) even when the person remains overweight or obese. For instance, in the course of treatment, someone may decide to focus primarily on physical activity (e.g., after experiencing its associated benefits for health and well-being), and be autonomously motivated for that outcome with only a small amount of weight loss, well below the recommended goal. Because in self-determination theory, promoting autonomy is a "non-negotiable" goal, those responsible for conducting obesity treatment programs may consider preserving or increasing participants' autonomy an equivalent outcome to that of weight loss. Indeed, recent evidence suggests that merely preserving (or not thwarting) autonomy may not be sufficient to ensure the development of autonomous motivation in the presence of competence and relatedness need support [33]. This may require a broader re-evaluation of the importance of psychological and behavioral in addition to biological health outcomes in obesity.

Patients and study participants are not the only ones who may experience pressure about achieving particular outcomes, with consequences for their motivation. If practitioners also feel pressure about achieving certain objective indicators of success, this could color their interactions with patients and inadvertently infringe on patient autonomy. This has previously been observed in tobacco cessation [34]. As recommended for practitioners in psychotherapeutic care [21], health professionals in obesity may also benefit from gaining clarity about their own motivations related to treatment, namely the extent to which they may feel controlled by external incentives (e.g., imposed by their health care organizations) or driven by internalized outcome contingencies, such as feeling that their own professional (and/or self-) worth is dependent on their patients' amount of weight loss. For the moment, empirical research is not available to either support or refute this hypothesis in obesity treatment. Nevertheless, having alternative indices of success (besides weight), such as increased self-efficacy and perceived autonomy around weight management, finding personal meaning in being physically active, or displaying a more flexible and positive relation to food and diet, as long as these are accepted as markers of success by participants, could help avoid these treatment traps.

#### Outcome-focused vs. process-focused treatment

A primary focus on weight - a number on a scale - as the single measure of success, while regarding exercise and diet primarily as a means to an end (weight loss) presents additional problems. First, it may tend to minimize the importance of the *process *of exercising, becoming physically fit, healthful eating, etc. and its inherent attributes and experiential elements, which could per se undermine behavior change [35]. Many sports and physical activities can clearly be a great source of enjoyment and provide a source of optimal challenge, to the point of being regulated primarily by intrinsic motivation [36]. A second limitation around a mostly instrumental view of lifestyle change is that whenever results do not meet initial expectations, take too long, or even *because *results are achieved, people may find themselves missing a good reason to continue their exercise and/or healthful eating efforts. A focus on fast results may even exacerbate these problems. For instance, more aggressive lifestyle changes (e.g., using a very low calorie diet or a reduced carbohydrate diet) are less likely to be explored for their inherent interest and instead valued only for their results. By contrast, an emphasis on making experiences worthwhile per se is not only clearly centered on the person's preferences but it also does not set inflexible boundaries or contingencies for success or failure. Indeed, there are indications that a dichotomous, all or nothing approach towards weight management as well as a rigid control of eating behavior negatively predict success [10,37]. From a self-determination theory perspective, rigid thinking and rigid behavioral patterns are thought to be maladaptive responses to conditions where basic needs are (or were) not satisfied [25]. They can provide an illusory sense of control, akin to what is described as introjected behavioral regulation, when preserving self-esteem and avoiding guilt are the primary energizers of behavior. As we will review later in this text, such controlled regulations are typically associated with less stable behavioral patterns[38,39].

#### Initial motivation

Another problem which could pervade weight control programs has to do with the initial motivation to lose weight on the part of overweight and obese individuals. The current environment seems to encourage extrinsic goals regarding body weight and physical appearance in general [40]. Not only can implicit social messages lead people, women in particular, to believe that thinness and attractiveness will automatically bring about happiness and well-being but obese people are also discriminated against in critical areas of life such as employment opportunities and health care [41]. Consequently, it is not surprising that people desire, and actually attempt, to lose weight in such large numbers [42]. Surprisingly, little research is available detailing motives for initiating a weight loss attempt or the impact of initial motivation on treatment outcomes [10]. One study in men found that health benefits, appearance, fitness, and well-being were the key reasons (in this order) mentioned for participating in a worksite weight management program [43], while another study with men and women reported that health (50%), appearance (35%), and improved mood (15%) were the "number one reason" to take part in a weight loss program [44]. Health (64%) and appearance (36%) were also given as the primary motives to start a "stop binge eating and lose weight program" [45]. Even considering that some people may not openly admit that body image improvement (i.e., appearance) is a central motive to lose weight, the previous findings suggest that a substantial number of individuals who attempt to lose weight have partially internalized pressuring forces and social constraints that value thinness above fatness. As noted above, self-determination theory researchers have proposed that some goals are considered extrinsic because meeting them is less fulfilling of basic psychological needs [25]. Aiming at social acceptance and status through physical appearance, or relying on motives associated with protecting self-worth and self-esteem (e.g., avoiding social discrimination) are unlikely to promote autonomous forms of motivation [24].

#### Beyond "behavior change"

Besides addressing how behaviors are regulated and integrated, self-determination theory is also concerned with human thriving, personal growth, and with the quality of (and vitality in) individuals' daily experiences [25]. Could there be a place in lifestyle change interventions to create the conditions for patients to strive for (intrinsic) goals beyond physical and mental health improvement, through behaviors such as cooking/eating, playing sports, or exercising? Could the internalization process in obesity behavioral treatment also be seen as the starting point for active self-actualization, for instance through learning new abilities and routines (e.g., becoming the cook of the house), relating to one's body in ways previously unknown (e.g., using dance as a form of personal and creative expression), contributing to one's community (e.g., by teaching others to be active and/or eat more healthfully), integrating new personal identities (e.g., becoming "a runner", who for the first time finishes a competitive popular race), or relating more closely to the natural world (e.g., in daily walks/runs in the local park or riverside path)? If autonomy and competence are recognized as human psychological nutriments at the most essential level, and if interventions are explicitly built to promote the fulfillment of those needs, then there is the possibility that individuals will thrive in that environment and reach a level of personal change beyond what is currently meant by "behavior modification". For instance, transference or spill-over effects in motivation and self-regulation from one behavioral domain to others are compatible with the principles of self-determination theory [46] and appear valid in its practice [47].

Whether long-lasting changes in eating and physical activity (and consequently in body weight) can in the future be reliably traced back to deeper personal transformative experiences is unknown [48]. Meanwhile, studies in the area of exercise and physical activity clearly show that perceived need support and autonomous forms of motivation are consistent predictors of behavior adoption and, in some cases, also maintenance [49,50]. The close association between regular exercise and long-term weight control [51] at least suggests that a similar pattern of associations could be observed more broadly in weight control. We review those data in the following section.

### Studies on autonomous motivation and weight control

Only a handful of studies have tested autonomy or other self-determination theory-related motivation variables as predictors of outcomes in the context of weight control interventions. Williams and colleagues [52] studied severely obese patients in the context of a 6-month medically-supervised very-low-calorie diet, where participants also received weekly group counseling intended to provide peer support, facilitate discussion, promote self-monitoring, etc. Perceived autonomy support and autonomous regulation were assessed immediately after the intervention and analyzed as predictors of exercise and weight loss. Results showed that perceived autonomy support predicted autonomous reasons to continue to participate in treatment, which in turn predicted higher attendance and improved weight loss. Path analysis supported these same mediation paths for outcomes at treatment end. Autonomous motivation at 6 months also correlated with self-reported exercise and weight loss at a 20-month follow-up [52]. Unfortunately, and despite the enormous growth in obesity studies in the recent 10-15 years [53], these encouraging findings did not spur immediate interest to "motivate" further research on the role of autonomous regulation in weight control.

**Figure 1:**
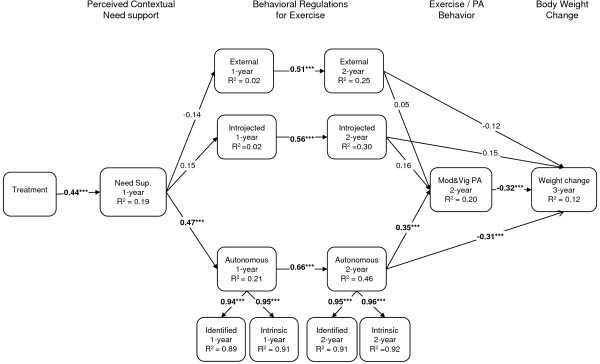
**Structural equation model (n = 135) for physical activity and weight change based on a self-determination theory-based 1-year weight control program**. Values in the paths represent the standardized bootstrap estimate for direct effects, *p < 0.05, **p < 0.01, ***p < 0.001 [39].

**Figure 2:**
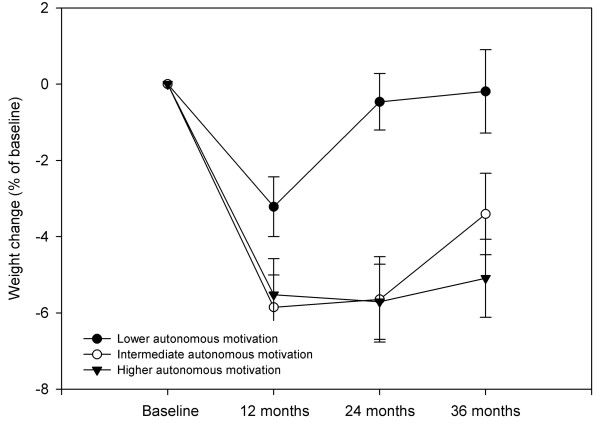
**Three-year weight change by tertile-split groups of exercise autonomous motivation, in initially overweight premenopausal women (n = 149)**. Between-groups differences in weight change (ANOVA) observed at 12 (p = 0.065), 24 (p < 0.001) and 36 months (p = 0.005). Autonomous motivation includes the *identified regulation *and *intrinsic motivation *subscales of the Exercise Self-Regulation Questionnaire. Values used for tertile-split groups were calculated at 24 months, including all subjects (intervention and control groups collapsed), adjusting for experimental group membership (see reference 39 for more details).

More recently, a randomized controlled trial was implemented to address the role of autonomous motivation in weight control, focusing on physical activity behaviors as well as on *motivation *for physical activity and exercise as putative mediating agents for long-term weight management, in premenopausal overweight and mildly obese women - the PESO study [54,55]. This intervention was found effective in changing perceived need support, need satisfaction, and autonomous regulation and intrinsic motivation for exercise, and also self-reported exercise and physical activity [54]. Subsequent analyses investigated the extent to which the hypothesized causal paths predicted exercise and weight outcomes immediately after the 1-year intervention and also at the 2- and 3-year follow-up assessments. One-year results supported a mediation effect of perceived need support and satisfaction of autonomy and competence needs for developing identified and intrinsic regulations for exercise. Autonomous exercise motivation was shown to mediate the effects of competence and autonomy on moderate and vigorous physical activity [54], a novel finding in overweight individuals. Exercise intrinsic motivation was a strong predictor of behavior change. In a subsequent study, three-year results confirmed the self-determination theory process model, showing that intervention-related changes in exercise autonomous regulation predicted 2-year self-reported moderate exercise and also 3-year weight control [39]. Figure 1 shows the structural model tested with parameter estimates and Figure 2 shows average weight changes at 3 years as a function of level autonomous exercise regulation 1 year after the intervention.

Within the same trial, an empirical test of a more diverse set of psychological and behavioral variables potentially associated with weight control showed that change in exercise motivation variables during the 12-month program (i.e., self-efficacy, perceived barriers, and intrinsic motivation) were significantly correlated with 2-year weight change [37]. A similar study had been conducted in US women who participated in a 4-month behavior weight control trial [56]. In post-hoc analyses, change in exercise intrinsic motivation was found to be the best predictor of 16-month weight changes, before and after weight loss during the 4-month program was controlled for in the model.

Also in the PESO study, Mata and colleagues analyzed whether global self-determination, motivation for staying in the trial, and exercise motivational regulations predicted successful eating self-regulation and mediated the association between actual physical activity and eating behavior [47]. Results were consistent with the *a priori *hypotheses and with the hierarchical model of motivation [57], suggesting that the quality of motivation could be one mechanism through which successful self-regulation in one area may spill-over into other behavioral domains. These findings could help explain how autonomously-motivated exercise behavior contributes to improved weight control; not only via the effects of physical activity itself, but also positively influencing the regulation of other relevant behaviors such as eating [58].

To our knowledge, only one study tested the association of eating self-regulation, investigated from a self-determination theory perspective (i.e., autonomous eating regulation) with changes in body weight [59]. Path analysis showed significant links between global motivation, self-determined motivation for healthful eating, actual dietary measures, and body weight. These findings are in line with the generalization of exercise motivation research findings to the regulation of eating behavior as described above [47]. They also support the hierarchical model of motivation from global *down to *eating-specific motivational regulation (top-down process). Although they did not measure weight control, Hagger et al., studied the association between perceived locus of causality for dieting and exercising, and intention and behavior in a cross-sectional sample of college students [60]. They found that autonomy predicted exercise intentions and behavior but it did not predict dieting behavior. Dieting behavior was assessed with a 2-item instrument, which could be an important limiting factor.

Finally, Gorin and colleagues (2008) investigated whether baseline levels of autonomous and controlled regulations and changes in regulations over 6 months were associated with 6-month weight outcomes in overweight women [61]. They found that higher controlled regulation at baseline was associated with less weight loss and that an increase in autonomous regulation and a decrease in controlled regulation over the 6-month period predicted more weight loss. A still ongoing study from the same team so far shows that autonomy support provided by other adults in the home environment leads to more autonomous regulation for weight control which in turn predicts larger weight loss (see [18]).

In summary, the available research linking self-determined motivation to weight control indicates a positive association between feelings of autonomy regarding healthful eating and especially physical exercise, and improved weight loss in the short and long-term. Some findings have highlighted a potential causal path from autonomy and autonomous motivation of behavior change for exercise and eating behavior, leading to improved weight control [39,47,52]. In addition to the perceived locus of causality and degree of autonomous regulation, evaluating exercise as intrinsically rewarding, interesting, and a source of enjoyment, as well as feeling confident towards implementing it also predicted successful weight control in other studies [37,56]. Conversely, in the studies reviewed above, no indication emerged that external and introjected regulation regarding exercise (or eating) is conducive to improved weight control, particularly in the long-term, despite the fact that interventions may induce increases in autonomous *as well as *in introjected regulations (e.g. [54]).

### Motivational Interviewing and weight loss programs

As discussed extensively elsewhere [62,63], MI and self-determination theory can be seen as complementary approaches to understanding behavior change and informing health-related interventions. MI is defined as a method of strengthening personal motivation for change [64]. Optimally implemented, one would expect MI to enhance individuals' autonomous motivation for change [65,66] (see also [63]). Thus research showing that MI interventions are effective in promoting healthy weight control could provide indirect evidence for the importance of autonomous motivation for successful behavior change. From our perspective then, with an emphasis on the need to promote autonomous motivation for change, it is encouraging that recently there have been a growing number of studies that have evaluated the effectiveness of MI in weight control programs. Although only two of the existing MI weight control studies have explicitly utilized self-determination theory as a theoretical framework [67,68], in most cases the authors explicitly state that MI was employed in recognition that the facilitation of internal or self-generated motivation for change is vital for sustained behavior change [9,69-72].

Studies have typically used MI as an adjunct to behavioral treatments or to standard care approaches (e.g., education on physical activity and diet) and the focus has been on weight control as the primary outcome variable. The results of this research are somewhat mixed. Five studies found an advantage for MI over comparison or control conditions [71-74]. Interestingly, Carels et al. [73] speculated that the observed weight loss benefits for an MI condition in their study could have been due to an increase in intrinsic motivation for behavior change. Unfortunately, motivation was not assessed in this study. One study found an advantage only for high attenders receiving a high intensity MI intervention [69]. Pollak and colleagues [75] found that physicians' use of MI-consistent techniques with overweight and obese patients predicted weight loss at three months whereas patients of physicians who used MI-inconsistent techniques gained or maintained weight.

Other studies have failed to show significant additional weight loss benefits for supplementing behavioral treatments with MI or in comparisons of MI with other interventions or control conditions [9,70,76-79]. However, in these studies the number of MI sessions was generally fewer than those with more positive outcomes. Furthermore, a number of studies have found advantages for MI in terms of weight-related outcomes other than weight loss, including physical activity [72,73], dietary behaviors [77], eating concerns and unrestrained eating [74], program adherence and glycemic control among type II diabetics [71,76], and a reduction in CHD risk factors [72]. Overall the evidence is at least suggestive that MI can be useful in weight control interventions but it remains unclear just how effective it is, and the extent to which it is effective in different populations [80].

Relatively few MI studies in weight control have included measures of motivation or considered motivation per se, and autonomous motivation in particular, as a desirable outcome to target or as a predictor or mediator of change. It is understandable that researchers should in the first instance be primarily interested in establishing treatment efficacy. Nevertheless, given the aforementioned explicit acknowledgement in many of the studies that the facilitation of internally-generated motivation for change is important for successful weight control, such studies offer an excellent opportunity to examine the motivational mechanisms underpinning successful, or indeed unsuccessful outcomes.

A number of well-validated instruments are available for assessing self-determination theory constructs which are suitable or adaptable for weight control contexts, including measures of perceived support for autonomy and the other psychological needs, satisfaction of psychological needs, and measures of autonomous and controlled regulation of behavior. Incorporating such measures in weight control interventions, and not just MI interventions, could help determine whether in comparison to control conditions (a) the intervention is perceived by participants as autonomy-supportive rather than controlling; (b) the intervention does lead to more autonomous motivation for engaging in adaptive weight control behaviors; and (c) enhancing autonomous motivation leads to greater adherence to adaptive behaviors and ultimately greater weight loss and maintenance. Adopting such measures could also help elucidate situations where interventions are found to be non-optimal or ineffective in producing desirable weight loss outcomes, for example by showing that the intervention led to more controlled rather than more autonomous motivation for change. For MI researchers in particular, even if they are not interested in self-determination theory per se, the use of such measures could provide support for the contention that MI is effective insofar as it promotes internal motivation for change and avoids externally coerced motivation.

Four studies so far have assessed autonomous motivation in MI interventions for weight loss (discussed below). All four used the Treatment Self-regulation Questionnaire (TRSQ: [52]) and they illustrate the usefulness of tassessing autonomous motivation in understanding intervention outcomes. In an intervention for weight loss among obese African American women, Befort et al. [9], compared a 16-week behavioral program with the addition of four sessions of MI with the same program with four sessions of didactic health education. Weight, dietary intake, physical activity, program adherence, and self-efficacy for diet and exercise were assessed, along with autonomous motivation. There was significant weight loss and improvements in diet in both groups but no differences between the two conditions, neither in autonomous motivation, self-efficacy, or program adherence. Furthermore and unexpectedly, autonomous motivation and exercise self-efficacy were significantly reduced in both groups, although post hoc analyses showed that higher motivation and self-efficacy at baseline were associated with greater decreases in motivation and self-efficacy. The authors proposed that the latter finding suggests that participants may have had unrealistic efficacy expectations and motivation at baseline.

Webber et al., [78] also assessed autonomous motivation in an 8-week internet-based intervention comparing MI with MI plus a discussion of values. Results showed a significant decrease in weight but no difference between groups. Autonomous motivation increased in both groups and, importantly, higher autonomous motivation at follow-up was associated with greater weight loss. Furthermore, the average number of self-motivating statements (change talk), expressed by participants during MI was correlated with an increase in autonomous motivation. An increase in change talk is considered the key 'active ingredient' in MI [64,81]. Thus, this study provides some evidence that MI can impact upon behavior change and weight outcomes by promoting autonomy.

Webber et al. [67] adopted SDT as a theoretical framework in comparing a standard internet-based weight loss program with a MI-based internet intervention among overweight or obese women, and assessed both autonomous and controlled motivation for weight loss. Although there was significant weight loss over 16 weeks, there was no difference between groups. However, examination of predictors of weight loss showed that treatment condition moderated the effect of baseline controlled motivation on weight loss. While low baseline controlled motivation was negatively associated with weight loss, moderation analysis revealed that individuals with high baseline controlled motivation lost less than 1 kg of body weight if they were assigned to the standard treatment group or 4.6 kg if they were assigned to a MI treatment group. Therefore, the MI-based intervention appeared to have buffered the negative effects of initial controlled motivation. This was confirmed by the similar weight loss results obtained among the individuals in the treatment group, whether they presented with high or low baseline controlled motivation. A different report from the same intervention has shown that motivational changes obtained during the first 4 weeks of treatment were associated with 16-week weight loss [67]. An increase in motivation, both autonomous and controlled, was observed during the 4 weeks. However, only individuals who obtained 5% weight loss or more at 16 weeks maintained the level of autonomous motivation throughout the treatment. The authors reported that the maintenance of autonomous motivation level was a possible mechanism by which the intervention might have affected adherence (measured by self-monitoring) and weight reduction.

West et al. [68], drawing partly on SDT as a theoretical framework, compared the effects of a novel motivation-focused approach, including an autonomy-promoting component based on MI, with a traditional skill-based approach on weight loss maintenance among overweight women. All participants underwent a six-month behavioral treatment program before being randomized to treatment conditions and followed for a further twelve months. The treatment conditions produced comparable sustained weight losses and both groups lost significantly more weight than controls. Participants in the motivational intervention group had significantly greater autonomous motivation for weight control than the skills-based group at the mid-point of the maintenance period, although the effect size was small. The authors suggested that motivational-based interventions are an effective alternative to available skill-based programs, and that future studies should investigate whether some women prefer one approach over the other [68].

A notable positive feature of the research on MI in weight control is that in most studies those delivering the interventions have been formally trained in MI and in the majority treatment fidelity checks have been conducted. On the other hand, the studies have tended to adopt or adapt selected discrete strategies from MI, combining them with alternative behavioral treatments, and the reports are often vague as to exactly how the interventions were implemented. In some studies, MI has been used principally to facilitate adherence to the behavioral treatments they have accompanied, or to enhance the efficacy of those treatments, rather than to promote autonomous motivation for engaging in a healthier lifestyle [9,74]. The potential problems with combining MI with other treatment approaches are lucidly discussed elsewhere [63] and we will not dwell on them here. Suffice it to say that reducing MI to a set of clinical techniques that are merely adjunctive to other treatments, or incorporating them into behavioral treatments that primarily emphasize outcomes (e.g., weight loss) that might themselves undermine autonomous motivation, carry the risk that any lasting benefits of MI may be reduced or eliminated.

## Summary and conclusions

In this article, we aimed to explore the topics of motivation and self-regulation from the viewpoint of self-determination theory, in the context of weight management and related behaviors. By doing so, we have offered a somewhat different perspective in the ongoing discussion around promoting sustained behavior changes in the broader literature, which represents one of the most difficult challenges facing health care professionals, behavioral scientists, and the individuals who struggle to make lifestyle change and manage their weight. Specifically, we aimed to i) critically address how motivation is viewed by current weight loss programs (whether explicitly or implicitly) and how that has translated into behavior change interventions and practices aiming at self-regulation, ii) present a complementary approach to understanding (self-) motivation for health behavior based on self-determination theory - including a consideration of autonomy in behavioral self-regulation, by analyzing the degree to which goals in obesity interventions are linked with the satisfaction of people's basic psychological needs, and more explicitly focusing on the *process *of behavior change relative to its (immediate) outcomes; iii) review empirical findings from weight control studies which have used measures of autonomy and/or the quality of motivation and analyze their association with weight outcomes, and iv) review studies that have evaluated MI in weight control programs. It was our premise that a consideration of qualitative dimensions of motivation is essential in helping individuals to be more successful in their efforts to achieve their goals, such as adopting physical activity or losing weight.

In weight control, as in other areas of behavioral medicine, we suggest that only to the degree to which individuals fully endorse behavioral goals and to the extent that the goals facilitate satisfaction of the needs for autonomy, competence, and relatedness will their efforts be more likely to result in behavioral change that is effectively maintained. Most interventions have thus far focused mainly on the "skills" or more functional aspects of behavior change (e.g., self-monitoring, problem-solving, contingency management) and may have relied too heavily on influencing people's cognitions, such as expectations about the immediate *outcomes *of their choices. In doing so, interventions may have commonly ignored important elements associated with the *process *involved in adopting new behaviors (e.g., developing genuine interest in exercise and physical activity or personal meaning in changing one's diet for good) and bypassed, or even undermined, what is necessary for effective and lasting internalization of new behaviors: curricula which explicitly support the development of autonomy (or "ownership") over the newly adopted behavioral patterns. As a consequence, participants and patients often appear dependent on actual weight loss for continuing to invest energy into treatment and/or they indicate that they need continuous support from health professionals. That is, the "whats" and "whys" of losing weight have been limited to extrinsic and mostly superficial aspects such as compliance with prescriptions, weight-contingent improvements in self-esteem, physical attractiveness, or the immediate gratification of a changing number on the scale. Moreover, goals have often been set out by health professionals, or indirectly promoted by society, but perhaps never entirely "validated" by (i.e., internalized into) the person's deeper sets of values and aspirations, with negative consequences for long-term self-regulation.

More promising, however, is the growth in studies employing self-determination theory-based interventions and/or MI in weight control programs. There are also exercise and eating behavior studies (see [82,83]), in some cases with overweight/obese participants, which have employed and/or tested the principles of self-determination theory for behavior change, with the aim of overcoming the traditional approach to motivation previously described. Generally, these studies have been supportive of the role of perceived autonomy and autonomous regulation in long-term behavior change. As to MI, the rationale for using it has often been that it has been shown to be effective in other behavioral domains. However, most researchers conducting these studies have also explicitly or implicitly acknowledged that motivation for change must come from within the individual [84]. Unfortunately, few of the studies have attempted to elucidate the mechanisms by which MI might exert its effects, or determine whether their interventions have actually promoted self-motivation. Future research in this area would benefit from drawing on self-determination theory to explore the motivational processes that mediate the effects of MI on successful treatment outcomes, including autonomous motivation and satisfaction of psychological needs [65]. A sounder understanding of these processes could allow us to refine and maximise the impact of MI interventions for weight control.

In conclusion, the current evidence is compatible with the notion that autonomous regulation is among the key predictors of successful weight outcomes. However, intervention research with obese individuals is still in the early stages. Meanwhile, the more abundant evidence for the role of autonomous and intrinsic motivation in exercise and physical activity adherence and studies that have analyzed autonomous eating self-regulation (both reviewed elsewhere [83], *Teixeira PJ, Carraça EV, Markland D, Ryan RM: Exercise, physical activity, and self-determination theory: A systematic review*. submitted) must serve as positive indicators, considering the fundamental role of these behaviors in weight management. More and better studies using MI in overweight/obese persons are also expected in the near future, to provide the needed experimental evidence that will definitively further our understanding of the role of motivation, and self-motivation, in long-term weight control.

## Competing interests

The authors declare that they have no competing interests.

## Authors' contributions

PJT led the writing of the manuscript and drafted the first version. DM led the writing of the section on Motivational Interviewing and revised the entire manuscript. JM, MNS, and ALP revised the entire text and made substantial contributions to subsequent versions. All authors read and approved the final manuscript.
